# Highly Efficient and Stable Organic Solar Cells via Interface Engineering with a Nanostructured ITR-GO/PFN Bilayer Cathode Interlayer

**DOI:** 10.3390/nano7090233

**Published:** 2017-08-23

**Authors:** Ding Zheng, Lili Zhao, Pu Fan, Ran Ji, Junsheng Yu

**Affiliations:** State Key Laboratory of Electronic Thin Films and Integrated Devices, School of Optoelectronic Information, University of Electronic Science and Technology of China (UESTC), Chengdu 610054, China; zd901023@gmail.com (D.Z.); 201511050104@std.uestc.edu.cn (L.Z.); fanpu1992@gmail.com (P.F.); jiran1231@gmail.com (R.J.)

**Keywords:** organic solar cell, reduced graphene oxide, PFN, bilayer cathode interlayer, in situ thermal reduction

## Abstract

An innovative bilayer cathode interlayer (CIL) with a nanostructure consisting of in situ thermal reduced graphene oxide (ITR-GO) and poly[(9,9-bis(3′-(*N*,*N*-dimethylamion)propyl)-2,7-fluorene)-alt-2,7-(9,9-dioctyl) fluorene] (PFN) has been fabricated for inverted organic solar cells (OSCs). An approach to prepare a CIL of high electronic quality by using ITR-GO as a template to modulate the morphology of the interface between the active layer and electrode and to further reduce the work function of the electrode has also been realized. This bilayer ITR-GO/PFN CIL is processed by a spray-coating method with facile in situ thermal reduction. Meanwhile, the CIL shows a good charge transport efficiency and less charge recombination, which leads to a significant enhancement of the power conversion efficiency from 6.47% to 8.34% for Poly({4,8-bis[(2-ethylhexyl)oxy]benzo[1,2-b:4,5-b′]dithiophene-2,6-diyl}{3-fluoro-2-[(2-ethylhexyl)carbonyl]thieno[3,4-b]thiophenediyl} (PTB7):[6,6]-phenyl-C_71_-butyric acid methyl ester (PC_71_BM)-based OSCs. In addition, the long-term stability of the OSC is improved by using the ITR-GO/PFN CIL when compared with the pristine device. These results indicate that the bilayer ITR-GO/PFN CIL is a promising way to realize high-efficiency and stable OSCs by using water-soluble conjugated polymer electrolytes such as PFN.

## 1. Introduction

Bulk heterojunction (BHJ) organic solar cells (OSCs) have drawn many interests in the past decades because of their suitability in the industrial manufacturing of large-area and flexible devices and in low-cost processing [1,2,3,4,5]. They also have a rapid energy payback time for sustainability and the development of clean energy [6]. The rapid progress of OSCs has recently yielded a remarkable power conversion efficiency (PCE) by exploiting interfacial engineering [7], device structure optimization [8], and rational material synthesis [9]. In particular, it has been demonstrated that interfacial engineering, such as inserting functional cathode interfacial layers (CILs) between the indium tin oxide (ITO) electrode and BHJ layer, plays a significant role in improving charge transportation and collection efficiency for inverted OSCs [10,11].

To date, a large number of materials have been utilized as the CILs in OSCs, such as *n*-type metaloxides (zinc oxide (ZnO), titaniumoxide (TiO*_x_*), aluminum oxide (Al_2_O_3_), etc.) [12,13,14,15], metallic compounds (ZnS, CdS, etc.) [16,17], and conjugated polymer electrolytes (CPEs; poly[(9,9-bis(3′-(*N*,*N*-dimethylamino)-propyl)-2,7-fluorene)-alt-2,7-(9,9-dioctylfluorene)] (PFN), etc.) [18,19,20]. Especially for PFN, it is found that the interlayer can improve the solar cell performance in multiple ways. First, CPE scan form appropriate dipoles at the electrode/active layer interface for turning the energy-level alignment of the electrode [19,21]. Second, CPEs improve the electron selectivity at the cathode by efficiently blocking holes [22]. However, there are several limitations of the utilization of PFN in further industrial applications of OSCs. First, a PFN layer that is too thick will act as an insulator because of its low conductivity, which dramatically increases the series resistance of the device, while a very thin layer of PFN (<10 nm) is usually required to serve as the effective surface modifier [23]. Additionally, because of the thin layer of PFN, the morphology is strongly dependent on the substrate it is spin-coated on. Usually, the morphology of PFN is rough and uneven, resulting from the rough surface of the ITO substrate [24]. Therefore, on account of these defects of PFN, it provides a limited improvement for the large-area fabrication and industrial application of OSCs. On the other hand, carbon-based materials, such as graphene, graphene derivatives (graphene oxide (GO) and reduced graphene oxide (R-GO)) [25,26], and carbon nanotubes (CNTs) [27,28], have been verified as excellent additives when used as the additive in the electron/hole extraction materials to improve the PCE [29,30]. Among these, R-GO is particularly attractive for applying to the CIL of OSCs because of its excellent properties, such as a high electric conductivity, a good soluble process ability, and excellent stability in ambient circumstances [31,32]. Therefore, further enhanced OSC performance and stability may be obtained by combining the advantages of PFN with those of R-GO. 

In this work, we introduce a novel bilayer CIL with a nanostructure that combines the R-GO template with PFN for high-performance and stable OSCs. By utilizing a spray-coating method [33], we can precisely control the film thickness of the GO template to obtain single- or multi-layer GO. Moreover, the sophisticated reduction of GO into R-GO by utilizing a facile one-step in situ thermal reduction (ITR) method is realized. Compared with pristine PFN, the ITR-GO/PFN bilayer structure serves as an efficient CIL with a more enhanced electrical conductivity. In addition, PFN spray-coated on single-layer ITR-GO (S-ITR-GO) can result in better energy-level alignment and a smoothed surface, thus enhancing charge-extraction efficiency and reducing recombination losses for the OSCs. As a result, by using the S-ITR-GO/PFN bilayer CIL, a significant enhancement in the PCE from 6.47% to 8.34% for a PTB7:PC_71_BM system has been obtained. The long-term stability of the OSC by using the ITR-GO/PFN CIL is also improved, when compared with the pristine device. 

## 2. Experimental Section

The GO powder was prepared from graphite powder (particle diameter of 45 nm; 99.99%; Sigma-Aldrich, St. Louis, MO, USA) according to the modified Hummers method [34]. Then, the obtained GO powder (20 mg) was dispersed in deionized water (10 mL). The suspension was ultrasonicated by utilizing a sonication system for 4 h in a water bath. The resultant suspension was centrifuged at 7500 rpm for 15 min to remove the aggregated GO particles. Finally, a dark yellow suspension of GO with a concentration of ~2 mg/mL was obtained for device fabrication. PFN solution was prepared by dissolving PFN material (99.9%; 2 mg; Sigma-Aldrich) and acetic acid (99.5%; 2 μL; Sigma-Aldrich) in methanol (99.8%; 2 mL; Sigma-Aldrich) under vigorous stirring for 12 h. The solution of PTB7:PC_71_BM (ratio of 1:1.5 wt %) in chlorobenzene (CB) with the addition of a small amount of 1.8-diiodooctane (DIO; 99.9%; CB:DIO = 97: 3 *v*/*v*; Sigma-Aldrich) with a total concentration of 20 mg/mL was used [35]. 

**Figure 1:**
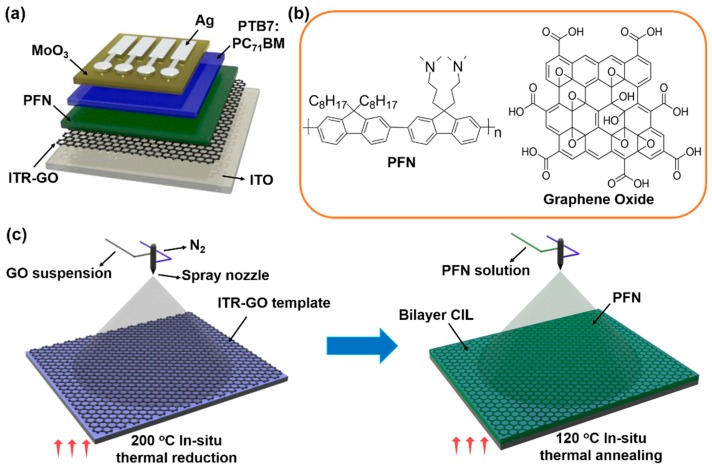
(**a**) Device architecture of organic solar cells (OSCs), (**b**) chemical structures of materials, and (**c**) schematic of fabrication process of bilayer cathode interlayer (CIL).

The chemical structures of the organic materials and the device architecture of the OSCs are shown in Figure 1a,b. The structure of the inverted OSCs was as follows: ITO/bilayer CILs (40 nm)/PTB7:PC_71_BM (100 nm)/MoO_3_ (15 nm)/Ag (100 nm). PTB7 (molecular weight: 80,000–200,000) and PC_71_BM were purchased from Solarmer Materials Inc. (Beijing, China). Prior to the processing of the functional layer, the ITO-coated glass substrates with a sheet resistance of 10 Ω/sq were consecutively cleaned in an ultrasonic bath containing detergent, acetone, deionized water and ethanol for 15 min at each step, and were then dried at 80 °C for 1 h. Then, the substrates were treated with ultraviolet/ozone (PSD-UV3, 40 W, NovaScan, Beijing, China) for 10 min. A layer of the ITR-GO template was spray-coated on the top of the ITO glass substrate at 200 °C by an air brush. The air brush was powered by N_2_ gas with a stream of 0.4 MP, and the injected flow rate of the GO suspension was 12 μL/s. The nozzle was 12 cm above the substrate. For the S-ITR-GO, the spray time was 3 s; for the multi-layer ITR-GO (M-ITR-GO), the spray time was 8 s. For the in situ thermal annealing treatment of the ITR-GO, we placed the substrate on a hot plate at 200 °C, and set the thermal annealing time equal to the spray time [29]. The schematic of the fabrication process of the bilayer CIL is shown in Figure 1c. Then, the PFN was spray-coated on the ITR-GO template. The air brush was powered by N_2_ gas with a stream of 0.6 MP, and the injected flow rate of the GO suspension was 15 μL/s. The nozzle was 12 cm above the substrate. The spray time of the PFN was 5 s for the ~15 nm thickness. For the in situ thermal annealing treatment of the PFN, we placed the substrate on a hot plate at 120 °C, and the thermal annealing time was also identical to the spray time (5 s). After that, the BHJ layer was spin-coated in the nitrogen box at 1500 rpm for 40 s without any annealing process. Then, a MoO_3_ (99.98%; Sigma-Aldrich) layer was deposited onto the substrates at a rate of 1–2 Å/s and at a pressure of 3 × 10^−3^ Pa in vacuum, which was followed by the deposition of the Ag anode at a rate of about 10 Å/s and under a pressure of 3 × 10^−3^ Pa, without breaking the vacuum.

The morphologies of the ITR-GO and CILs were obtained by using atomic force microscopy (AFM; Agilent, Santa Clara, CA, USA, AFM 5500). X-ray photoelectron spectroscopy (XPS) and ultraviolet photoelectron spectroscopy (UPS) were performed by a Thermo Scientific ESCALAB 250Xi instrument (dual source, Waltham, MA, USA). Spectra were obtained after the surface of the film was etched to about 2 nm to minimize surface contamination. Raman spectroscopy data were collected by the Acton TriVista Confocal Raman Spectroscopy System (Santa Clara, CA, USA). The current density–voltage (J-V) characteristics of the BHJ OSCs were measured with a simulated light source (CHF-XM35, Beijing Trusttech, Beijing, China) with an illumination power of 100 mW/cm^2^. The electrical data were recorded using a Keithley 4200 source-measure unit (Cleveland, OH, USA). External quantum efficiency (EQE; SolarCellScan 100, Zolix, Wuhan, China) spectra were measured under the lump light passing through a monochromator, which was calibrated by a standard silicon solar cell. 

## 3. Result and Discussion

### 3.1. Fundamental Properties of the ITR-GO Template

**Figure 2:**
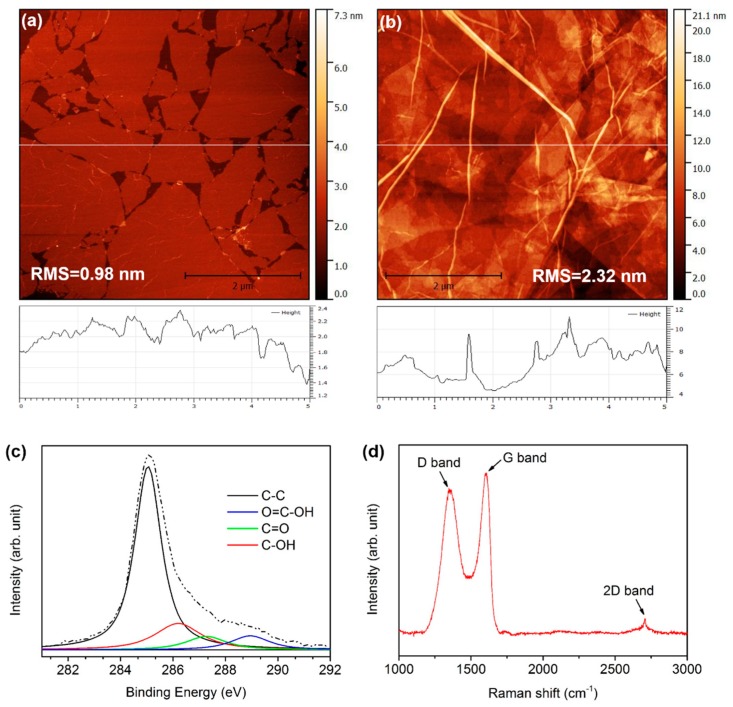
Atomic force microscopy (AFM) images of single-layer in situ thermal reduction grapheme oxide(S-ITR-GO) (**a**) and multi-layer ITR-GO (M-ITR-GO) (**b**); Raman spectrum (**c**) and X-ray photoelectron spectroscopy (XPS) spectra (**d**) of ITR-GO.

AFM was utilized to characterize the morphology of the S-ITR-GO (Figure 2a) and M-ITR-GO (Figure 2b). The thickness of the S-ITR-GO was~1.04 nm, which matched well with the thickness of a single-layer GO sheet [36]. When we increased the spray time, the S-ITR-GO overlapped to form M-ITR-GO. From the AFM image, the rough and thick morphology of the M-ITR-GO can be observed. The chemical structures and elementary composition of the ITR-GO were measured by XPS. Figure 2c illustrates the curve fittings of the C1s peak of the XPS spectra of the spray-coated GO and spray-coated ITR-GO films. A binding energy of 285.1 eV is attributed to C–C/C–H bonds. The peaks which are centered at the binding energies of 286.3, 287.3 and 289.0 eV are assigned to C–OH, C=O, and O=C–OH functional groups, respectively [37,38,39]. For the ITR-GO film, the peak at 287.3 eV (C=O bonds) and the peak at 289.0 eV (O=C–OH bonds) nearly disappear. Thus, the decreased concentration of oxygen-containing functional groups after ITR shows that the ITR treatment was an effective and facile reduction method for the GO film. Raman spectra were also obtained to characterize the ordered/disordered crystal structures and natural characteristics of the spray-coated ITR-GO films (Figure 2d). It is well known that the G band corresponds to the first-order scattering of the E_2g_ mode related to the vibration of sp^2^-bonded carbon atoms, and the D band, to a breathing mode of point phonons of A_1g_ symmetry [40], which is assigned to defects and disorders, especially at the edges of graphene [41]. The spray-coated ITR-GO film shows a D band at 1354 cm^−1^ and a G band at 1603 cm^−1^ with an intensity of d peak versus intensity of g peak (ID/IG) of 0.89. The ID/IG reveals the level of disorder, as expressed by the sp^3^/sp^2^ carbon ratio. In this case, ITR-GO has a depressed D band peak intensity and a smaller ID/IG ratio value. As a result, the ITR process can reduce the defect quantity of graphene. 

### 3.2. Characterization of ITR-GO/PFN Bilayer CILs

**Figure 3:**
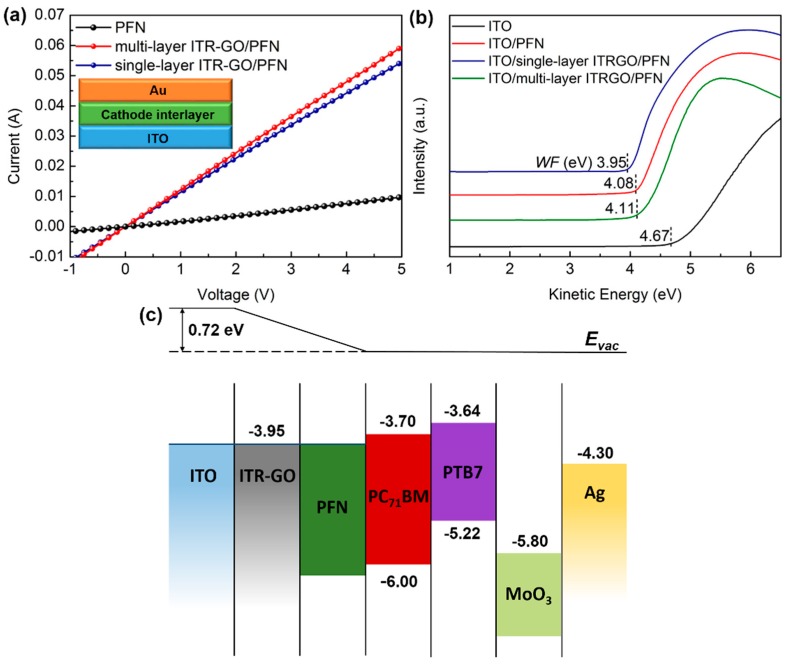
(**a**) Current-voltage curves of the organic solar cells (OSCs) for ITO/CILs/Au. (**b**) Ultraviolet photoelectron spectroscopy (UPS) cutoff of ITO substrates with various layers on top. (**c**) Schematic energy levels of the inverted device.

The current-voltage features of CIL films (ITO/CIL/Au) are displayed in Figure 3a, and the conductivity values of CIL films are listed in Table 1. The conductivity values of PFN, S-ITR-GO/PFN, and M-ITR-GO/PFN were calculated as 5.72 × 10^−3^ S/cm, 3.32 × 10^−2^ S/cm and 3.60 × 10^−2^ S/cm, respectively. It is apparent that the bilayer CIL of ITR-GO/PFN exhibits a large enhancement in conductivity when compared with bare PFN. The enhanced electrical conductivity is attributed to the incorporation of highly conductive ITR-GO. This increased conductivity ofthe bilayer CIL is beneficial for restraining charge recombination and for facilitating electron extraction in OSCs.

Meanwhile, UPS was used to investigate the work function (WF) change on ITO substrates with PFN and ITR-GO/PFN modification. As shown in Figure 3b, the WF shifted from 4.67 eV to 4.08 eV upon ITO modification with PFN. The 0.59 eV shift of the ITO WF suggests that PFN turns the WF by the formation of appropriate dipoles at the interface between the electrode and active layer. The WF of ITO also shifted from 4.67 eV to 3.95 eV and 4.11 eV with the modification of S-ITR-GO and M-ITR-GO/PFN CIL, respectively. These results indicate that the ITR-GO template between ITO and PFN did not affect the formation process of the dipoles. The ITR-GO/PFN bilayer CIL could also reduce the WF of ITO to increase the built-in potential, leading to the reduction of energy offsets for the energy alignment of the OSCs [42]. The schematic of the energy levels is displayed in Figure 3c.

**Figure 4:**
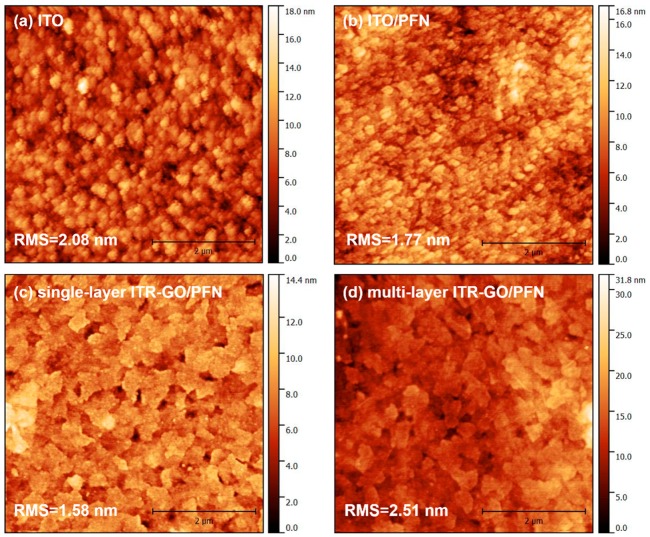
(**a**) Atomic force microscopy (AFM) images of ITO, (**b**) poly[(9,9-bis(3′-(*N*,*N*-dimethylamion)propyl)-2,7-fluorene)-alt-2,7-(9,9-dioctyl) fluorene] (PFN) on ITO, (**c**) single-layer in situ thermal reduction graphene oxide (S-ITR-GO)/PFN on ITO, and (**d**) multi-layer ITR-GO (M-ITR-GO)/PFN on ITO.

To better understand the morphologies of PFN sprayed on the ITR-GO template, AFM measurements were taken, as shown in Figure 4. The AFM height images of ITO and PFN spray-coated on ITO are shown in Figure 4a,b, respectively. It can be seen that the domain size of the PFN spray-coated on ITO was almost the same as that of ITO. The root mean square (RMS) of the roughness decreased from 2.08 to 1.77 when PFN was spray-coated on ITO. This result indicates that PFN can modify the surface of ITO, and provide a smoother interface between the active layer and the electrode. 

Furthermore, to investigate the morphology of PFN layers spray-coated on ITR-GO templates, AFM height images of PFN spray-coated on S-ITR-GO and M-ITR-GO were characterized and are shown in Figure 4c,d, respectively. Compared with the image of ITO and PFN spray-coated on ITO, the domain sizes of PFN significantly increased on the top of both the S-ITR-GO and M-ITR-GO templates. As distinguished from the granular shape of the PFN domains spray-coated on ITO, the flake-like domains of PFN spray-coated on ITR-GO are formed. Thus, we can see that the morphology of PFN is strongly affected by the substrate or template. Nevertheless, the RMS roughness of the flake-like PFN on S-ITR-GO and M-ITR-GO is quite different. The PFN on the top of the M-ITR-GO template is much rougher than that on the S-ITR-GO template. The result shows that the roughness of PFN also depends on the template’s roughness, and the S-ITR-GO template can offer a smoother surface for PFN coated on it. 

### 3.3. Device Performance of OSCs Based on Various CILs

**Figure 5:**
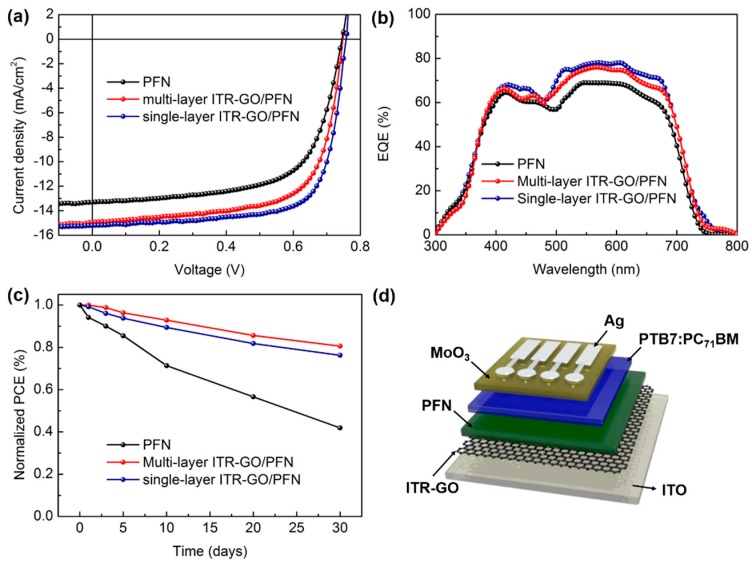
(**a**) Current density-voltage (J-V) curves of organic solar cells (OSCs) with different cathode interlayer (CILs). (**b**) External quantum efficiency (EQE) of OSCs with different CILs. (**c**) Stability of OSC devices in ambient air without encapsulation. (**d**) Device architecture of OSC.

To further illuminate the influence of various CILs on device performance, OSCs with various CILs were fabricated. Their typical J-V curves are presented in Figure 5. The detailed characteristics of devices with different CILs, including the open-circuit voltage (*V_OC_*), current density (*J_SC_*), fill factor (FF), and PCE, are listed in Table 1. The device based on pristine PFN yielded a relatively low PCE, *J_SC_*, *V_OC_* and FF of 6.47%, 13.31 mA/cm^2^, 0.75 V and 64.87%, respectively. The *J_SC_* of the OSC increased from 13.31 mA/cm^2^ to 14.92 mA/cm^2^, and the FF increased from 64.87% to 67.90%, when using M-ITR-GO/PFN as the bilayer CIL. The device composed of an M-ITR-GO/PFN bilayer CIL had a PCE that increased from 6.47%to 7.59%. Particularly, the OSC based on an S-ITR-GO/PFN bilayer CIL also showed an increased *J_SC_*, *V_OC_*, FF, and PCE of 15.21 mA/cm^2^, 0.76 V, 72.16% and 8.34%, respectively, leading to the best PCE of the devices.

The improvement in the *J_SC_* value was mainly due to the increased conductivity values of the bilayer CIL and larger PFN domains, which allows the photogenerated carriers to propagate through the device rapidly and avoid the defects [24]. The increased *V_OC_* indicates a good charge transfer between the active layer and buffer layer of the fabricated device. This phenomenon can be attributed to the better energy-level alignment at the CIL and active interface. The S-ITR-GO/PFN bilayer CIL had a suitable energy level of 3.92 eV for its corresponding interface, resulting in the improved *V_OC_* value. These highly conductive modified CILs also facilitate the charge collection and reduce the charge recombination in OSCs. Moreover, the single-layer template could modify the PFN surface, leading to a smooth surface associated with a large domain size to suppress charge trapping and recombination [43]. These three factors played the key role for the increased FF when using the S-ITR-GO/PFN bilayer CIL. Clearly, the OSC consisting of the S-ITR-GO/PFN bilayer CIL exhibited a higher FF and *V_OC_* than the device based on the M-ITR-GO/PFN bilayer CIL, indicating that a smoother surface of the PFN can facilitate the interfacial contact between the CIL and the active layer. Ultimately, the OSC with the S-ITR-GO/PFN bilayer CIL yielded a high FF and *V_OC_*, and thus had the best PCE of 8.34%. 

The EQE spectra with various CILs are shown in Figure 5b, showing that the photon responses of devices composed of an ITR-GO/PFN CIL were higher than those of the pristine device, which ranged from 400 nm to 800 nm. The calculated *J_SC_* values from the EQE of the pristine device, and the devices based on S-ITR-GO and M-ITR-GO/PFN bilayer CILs were 13.12, 15.01, and 14.51 mA/cm^2^, respectively, which was in good agreement with the measured *J_SC_* value. The high EQE originated from the high conductivity of the ITR-GO/PFN CIL increasing the electron transport efficiency.

To study the air ambient stability of the OSCs using various CILs, the devices were tested without encapsulation, and the device performances with different storage times are shown in Figure 5c. The PCE of the devices based on pristine PFN dropped to 50% after 30 days, which shows the poor stability. In contrast, the devices based on ITR-GO/PFN CILs showed a higher ambient stability; particularly, the device based on M-ITR-GO/PFN CILs retained 85% of its original PCE after being stored in air ambient for 30 days. Consequently, the OSC using ITR-GO/PFN bilayer CILs exhibited excellent efficiency as well as an improved stability compared to pristine PFN-based OSCs.

## 4. Conclusions

In summary, we successfully demonstrated that inverted OSCs had a dramatic enhancement of their PCE and stability by introducing a nanostructured ITR-GO/PFN bilayer CIL processed by a spray-coating method. The one-step ITR treatment, which reduces GO into ITR-GO, can significantly improve the electrical conductivity of the ITR-GO/PFN bilayer CIL. Moreover, this bilayer CIL has other excellent properties, including a smooth nanostructure thin film and a suitable energy level for charge transfer. The bilayer CIL for inverted OSCs can boost the electron transport and extraction between the cathode and the active layer, and can reduce the carrier recombination accordingly. The OSC consisting of the ITR-GO/PFN CIL had a PCE that increased from 6.47% to 8.34%, and the long-term stability was also improved by the ITR-GO/PFN CIL. This result shows that the formation of nanostructured ITR-GO/PFN bilayer CILs by combining ITR-GO with PFN is an effective way to avoid the defects of PFN or other CPEs. The fabrication process by the spray-coating method also provides a facile means to realize high-performance and stable OSCs, and it has high potential for large-scale manufacturing and application versatility. 

## Figures and Tables

**Table 1:** Detailed characteristics of organic solar cells (OSCs) based on various cathode interlayers (CILs). Average values and standard deviations are deduced from 20 devices.

CILs	Conductivity (S/cm)	WF (eV)		*V_OC_* (V)	*J_SC_* (mA/cm^2^)	FF (%)	PCE (%)
ZnO [29]				0.74	−13.69	65.94	6.68
PFN	5.72 × 10^−3^	4.08	Average	0.74	−13.02	64.45	6.21 ± 0.31
			Best	0.75	−13.31	64.87	6.47
M-ITR-GO/PFN	3.60 × 10^−2^	4.11	Average	0.75	−14.54	66.36	7.24 ± 0.12
			Best	0.75	−14.92	67.90	7.59
S-ITR-GO/PFN	3.32 × 10^−2^	3.95	Average	0.76	−15.082	69.97	8.02 ± 0.24
			Best	0.76	−15.21	72.16	8.34
